# From Routine Supplementation to Targeted Repletion: Vitamin D Deficiency in UVB-Constrained, Pollution-Exposed, and Ageing Populations—Musculoskeletal and Extra-Skeletal Perspectives

**DOI:** 10.3390/nu18162736

**Published:** 2026-08-21

**Authors:** Yonghyun Yoon, Jihyo Hwang, Jungyoun Kim, Ki-Tae Kim, Chan-Mo Yang, Rowook Park, Jaehyun Shim, Jonghyeok Lee, Seungbeom Kim, Youngmo Kim, King Hei Stanley Lam

**Affiliations:** 1 Department of Orthopaedic Surgery, Hallym University Kangnam Sacred Heart Hospital, 1 Singil-ro, Yeongdeungpo-gu, Seoul 07441, Republic of Korea; 2 Incheon Terminal Orthopedic Surgery Clinic, 489beon-gil, Inha-ro, Namdong-gu, Incheon 21574, Republic of Korea; 3 International Academy of Regenerative Medicine, 489beon-gil, Inha-ro, Namdong-gu, Incheon 21574, Republic of Korea; 4 Department of Psychiatry, Wonkwang University Hospital, Iksan 54538, Republic of Korea; 5 Sae Yonsei Rehabilitation Clinic, Seoul 06972, Republic of Korea; 6 Chungdammadi Neurosurgery Clinic, Seoul 06099, Republic of Korea; 7 Bareun Neurosurgery Clinic, Cheongju 28424, Republic of Korea; 8 Miso Pain Clinic, Suwon 16703, Republic of Korea; 9 Faculty of Medicine, The University of Hong Kong, Hong Kong, China; 10 Faculty of Medicine, The Chinese University of Hong Kong, New Territories, Hong Kong, China

**Keywords:** 25-hydroxyvitamin D, osteomalacia, cholecalciferol dosing, musculoskeletal health, osteoporosis, risk-stratified care, narrative review

## Abstract

**Background/Objectives:** Vitamin D deficiency remains common in UVB-constrained, pollution-exposed, sedentary, and ageing populations, yet recent guidelines discourage routine screening and empiric high-dose supplementation in generally healthy adults. This review clarifies the clinical distinction between untargeted population supplementation and monitored repletion of documented deficiency in high-risk individuals. Methods: We conducted a focused narrative review of PubMed/MEDLINE, Scopus, Web of Science, and the Cochrane Library. The focused evidence search supporting the narrative synthesis was completed in June 2026. References published subsequently were included during manuscript preparation only when relevant for contextual purposes and were not incorporated retrospectively into the evidence-selection process underlying the review’s clinical conclusions. Evidence was interpreted according to baseline and achieved 25-hydroxyvitamin D [25(OH)D], dosing schedule, route, body composition, endpoint, and population risk. No formal risk-of-bias assessment, certainty grading, or systematic meta-analytic synthesis was performed. **Key Findings:** The strongest rationale for vitamin D repletion remains musculoskeletal, particularly for osteomalacia, muscle function, osteoporosis care, and fracture-related vulnerability in deficient or high-risk patients. Management should separate generally healthy adults from patients with an indication for testing or treatment. Historical and regional expert frameworks describe time-limited higher-dose repletion for documented deficiency, but these regimens should not be interpreted as a current universal Endocrine Society treatment standard. Short-term trials using moderate cumulative oral doses suggest broadly comparable biochemical repletion with daily, weekly, or monthly schedules; equivalence has not been established for long-term clinical outcomes, frail populations, or large intermittent doses. Extra-skeletal associations remain less trial-supported. Conclusions: Vitamin D deficiency should be managed using clinically indicated assessment, explicit repletion and maintenance regimens, and selective biochemical reassessment. Targets above conventional skeletal adequacy thresholds remain investigational and should not be applied as universal treatment goals.

## 1. Introduction

Vitamin D deficiency remains a prevalent nutritional problem in populations exposed to converging geographic, environmental, behavioural, physiological, and dietary constraints, including seasonally constrained ultraviolet-B exposure, air pollution, indoor living, photoprotective behaviour, ageing, and limited dietary intake. Although vitamin D is classically defined by its role in calcium-phosphate homeostasis and skeletal mineralisation, vitamin D receptor signalling is also involved in immune regulation, vascular biology, cellular differentiation, and neurotransmitter-related pathways. These extra-skeletal associations have generated substantial clinical interest, but their interpretation remains inconsistent across specialties. Clinicians in orthopaedic, nutritional, cardiovascular, oncological, and psychiatric settings often evaluate the same deficiency state using different thresholds, endpoints, and assumptions about clinical benefit [1,2,3].

This narrative review evaluates vitamin D deficiency as a multisystem nutritional risk while maintaining a clear distinction between biological plausibility, observational association, and trial-supported clinical benefit. The review is structured around three questions: first, why vitamin D deficiency persists in modern UVB-constrained and urbanised populations; second, how deficiency relates to musculoskeletal and selected extra-skeletal outcomes; and third, how supplementation evidence should be interpreted when large trials of untargeted supplementation have often been neutral. Particular attention is given to the distinction between population-wide supplementation and targeted correction of documented deficiency, and to how baseline 25-hydroxyvitamin D [25(OH)D] status, achieved concentration, body composition, dosing schedule, route of administration, and clinical endpoint may influence trial interpretation [4,5,6]. The specific contribution of this review is therefore not a new definition of deficiency, but an evidence-interpretation framework that separates population-level prevention questions from clinically indicated management of established deficiency and explicitly maps the differing evidentiary strength of skeletal and extra-skeletal outcomes.

South Korea serves as a regional case context because it illustrates several determinants that are increasingly relevant worldwide: northern-temperate latitude, dense urban living, particulate air pollution, seasonal Asian dust exposure, widespread photoprotection, indoor occupations, sedentary behaviour, and rapid population ageing. KNHANES analyses have documented a high prevalence of low 25(OH)D, with particularly low concentrations among young adults, an age group during which peak bone mass continues to consolidate. The Korean example is therefore not used to make country-specific causal claims, but to illustrate how environmental, behavioural, and demographic pressures can converge to sustain vitamin D deficiency in modern societies [2,3,7,8].

Comparable population-level data from other regions support an international rather than exclusively Korean perspective, while also showing important geographic heterogeneity. Standardised European data have demonstrated a substantial prevalence of low 25(OH)D across multiple countries, whereas Canadian Health Measures Survey data show generally higher population status but persistent inadequacy in specific demographic groups. Cross-continental reviews of national nutrition surveys further highlight major differences in sampling, biomarker collection, and survey design that limit direct country-to-country comparisons. Accordingly, South Korea is used here as an illustrative regional case rather than as a surrogate for all populations [9,10,11].

Previous integrative work has described vitamin D as a multifunctional molecule with broad skeletal and extra-skeletal relevance [12]. In contrast, the present review addresses a narrower clinical problem: how clinicians should interpret and manage documented vitamin D deficiency in populations in whom ultraviolet-B exposure is structurally constrained by latitude, season, pollution, indoor living, photoprotection, skin pigmentation, and ageing. This distinction is central. The absence of clear benefit from routine supplementation in unselected, largely replete populations is not equivalent to evidence against targeted correction of genuine deficiency in metabolically, musculoskeletally, or nutritionally vulnerable patients.

## 2. Methods

### 2.1. Review Design and Objectives

This article was designed as a narrative review of vitamin D deficiency and repletion in clinical nutrition practice, informed by established principles for transparent narrative review conduct and reporting. The aim was not to perform a quantitative synthesis of a single clinical endpoint, but to integrate mechanistic, epidemiological, interventional, and guideline-based evidence across musculoskeletal and extra-skeletal domains [13,14]. The review was structured around five clinical questions: (1) which environmental, behavioural, and demographic factors contribute to vitamin D deficiency in UVB-constrained and ageing populations; (2) which musculoskeletal outcomes are most consistently linked to deficiency; (3) how strong is the evidence for cardiovascular, immunological, oncological, and neuropsychiatric associations; (4) how should neutral randomised supplementation trials be interpreted in relation to baseline vitamin D status, dosing schedule, body composition, and endpoint selection; and (5) which practical repletion strategies are supported for selected individuals with documented deficiency.

### 2.2. Literature Search

A focused literature search was conducted in PubMed/MEDLINE, Scopus, Web of Science, and the Cochrane Library. The focused evidence search supporting the narrative synthesis was completed on 30 June 2026. Search terms combined vitamin D-related keywords, including “vitamin D deficiency,” “25-hydroxyvitamin D,” “25(OH)D,” “cholecalciferol,” “calcifediol,” and “1,25-dihydroxyvitamin D,” with domain-specific terms related to latitude, ultraviolet-B exposure, air pollution, behaviour, musculoskeletal outcomes, cardiovascular disease, cancer, immune outcomes, neuropsychiatric outcomes, dosing, route, toxicity, and clinical guidance. Reference lists of major guidelines, systematic reviews, meta-analyses, individual-participant-data meta-analyses, and landmark randomised trials were also reviewed to identify additional relevant studies. References published after the search cutoff could be cited during manuscript preparation when they served a contextual purpose, including citation of the authors’ related work; such citations were not treated as search-derived evidence and did not alter the evidence hierarchy or clinical conclusions. Exact database-specific executable search strings were not prospectively archived because the review was conducted as a focused narrative synthesis rather than a prospectively registered systematic review; we therefore report the databases, final search date, search domains, and source-selection process rather than retrospectively reconstructing search strings that could imply greater reproducibility than was available.

### 2.3. Source Selection

Sources considered for the review included clinical practice guidelines, position statements, randomised controlled trials, individual-participant-data meta-analyses, systematic reviews and meta-analyses, large population-based cohort studies, Mendelian randomisation studies, pharmacokinetic studies, and mechanistic studies with direct relevance to human vitamin D physiology. Studies were prioritised when they addressed baseline vitamin D status, achieved 25(OH)D concentration, dosing schedule, formulation, route of administration, clinically meaningful endpoints, or populations relevant to deficiency risk. The evidence base relied predominantly on publications that could be directly verified by the review team, primarily English-language full texts. Korean and East-Asian studies were included when directly relevant to the regional case context, but geographical relevance alone was not considered sufficient for inclusion.

The search was intended to support a focused narrative synthesis rather than exhaustive systematic identification. Two reviewers assessed candidate articles through title/abstract review followed by full-text evaluation of directly relevant sources, with differences in inclusion judgment resolved by discussion. Key claims, numerical data, and their supporting citations were independently cross-checked by three authors. Because a prospective deduplicated screening log was not maintained for this narrative review, exact PRISMA-style record counts cannot be reconstructed reliably and are therefore not reported. No formal risk-of-bias tool or certainty-of-evidence grading system was applied.

Lower priority was given to narrative commentaries without primary data, non-peer-reviewed preprints without subsequent publication, small uncontrolled case series, studies with unclear vitamin D formulation or dosing, studies relying primarily on surrogate outcomes of uncertain clinical relevance, and reports superseded by larger or more recent systematic reviews. Retracted studies were not used as supporting evidence; when mentioned, they were discussed only to clarify why a particular line of evidence should not be relied upon.

### 2.4. Evidence Prioritisation and Interpretation

Evidence was interpreted hierarchically according to clinical domain. For skeletal outcomes, priority was given to randomised trials, fracture and fall meta-analyses, osteoporosis guidelines, and studies of severe deficiency, osteomalacia, sarcopenia, and hip fracture outcomes. For cardiovascular disease and atherosclerosis, mechanistic plausibility and observational associations were considered separately from randomised supplementation trials and Mendelian randomisation evidence. For cancer and immune outcomes, cancer incidence was distinguished from cancer mortality, and daily dosing was distinguished from intermittent bolus regimens. For neuropsychiatric, sleep, and pain-related outcomes, observational and mechanistic evidence was interpreted as hypothesis-generating unless supported by randomised trial data.

Causal language was reserved for outcomes supported by randomised or genetic evidence. Observational and mechanistic findings were described as associations, biological plausibility, or vulnerability models. Null primary-prevention trials were interpreted according to the populations and interventions studied, including baseline vitamin D status, population selection, adiposity, endpoint latency, achieved 25(OH)D concentration, formulation, and dosing schedule.

### 2.5. Synthesis Approach

Because this review spans multiple clinical domains and does not address a single PICO question, no formal meta-analysis was performed. A PRISMA flow diagram and formal risk-of-bias assessment were not applied, as the objective was not to generate pooled effect estimates but to provide a clinically oriented interpretation of heterogeneous evidence. Instead, findings were synthesised narratively using a domain-based framework: determinants of deficiency; musculoskeletal consequences; cardiovascular and atherosclerotic evidence; immunological and oncological implications; neuropsychiatric, sleep, and pain-related associations; interpretation of neutral supplementation trials; therapeutic targets; route and dosing strategy; and public-health implications.

To improve transparency, the synthesis was organised around guiding review questions, clinically relevant evidence domains, transparent source-selection principles, and qualitative interpretation of evidence strength, consistent with recommended principles for narrative review reporting. The strength of inference was characterised according to study design, consistency of findings, clinical endpoint relevance, population applicability, and concordance between mechanistic, observational, and interventional evidence [13,14]. SANRA domains were used as a reporting and quality-structuring aid rather than as a formal scored instrument, and no numerical SANRA score was used to determine study inclusion or evidence certainty.

## 3. The Latitude–Environment–Behaviour Triad

Vitamin D status reflects the balance between cutaneous synthesis, dietary intake, absorption, distribution, and metabolic activation. In UVB-constrained, urbanised, photoprotective, sedentary, and ageing populations, several determinants act in the same unfavourable direction. This section considers five interacting drivers: latitude-related ultraviolet-B insufficiency, atmospheric attenuation by air pollution and dust, behavioural photoprotection, sedentary indoor lifestyles with loss of incidental sunlight exposure, and ageing-related decline in cutaneous synthesis [1,8,15,16].

Because no single serum 25(OH)D threshold is universally endorsed across current frameworks, the present review does not apply one universal deficiency cutoff across all clinical domains; threshold interpretation is discussed in detail in Section 9.

**Figure 1:**
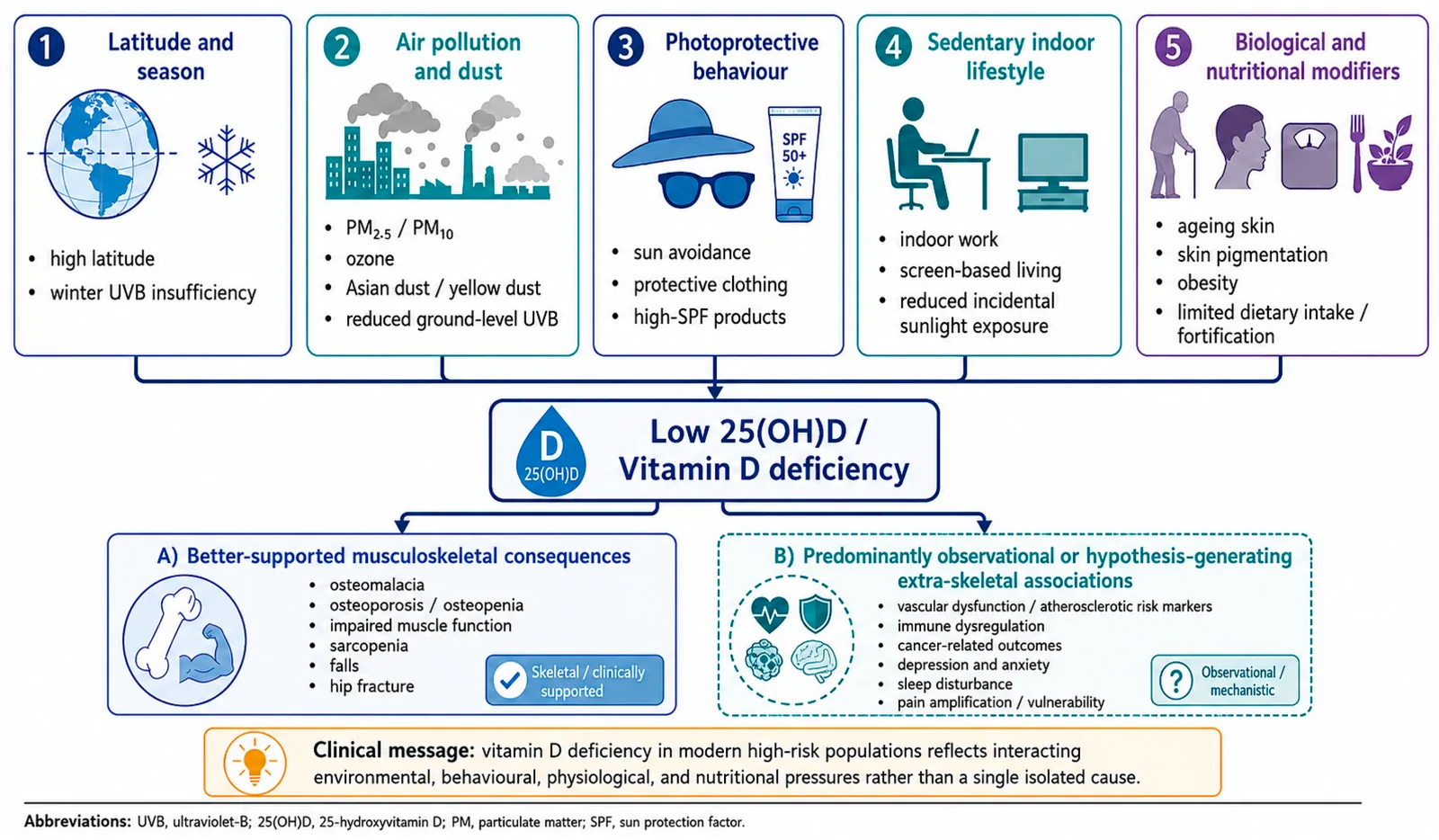
Determinants and clinical implications of vitamin D deficiency. Environmental, behavioural, physiological, and nutritional determinants converge to reduce serum 25-hydroxyvitamin D [25(OH)D], including latitude and season, air pollution and dust exposure, photoprotective behaviour, sedentary indoor lifestyle, ageing skin, skin pigmentation, obesity, and limited dietary intake or fortification. Musculoskeletal consequences are comparatively well established, whereas the extra-skeletal panel represents predominantly observational, mechanistic, or hypothesis-generating associations and should not be interpreted as a causal treatment pathway. Abbreviations: 25(OH)D, 25-hydroxyvitamin D; PM, particulate matter; SPF, sun protection factor; UVB, ultraviolet-B. Generated using ChatGPT Images 2.0 (OpenAI, 2026) from author-specified scientific content; the final figure was critically reviewed and approved by all authors for scientific accuracy.

These interacting determinants and their downstream musculoskeletal and extra-skeletal implications are summarised in Figure 1.

### 3.1. Latitude and Ultraviolet-B Insufficiency

Cutaneous synthesis of vitamin D3 requires ultraviolet-B (UVB) photons in the 290–315 nm band to convert 7-dehydrocholesterol to previtamin D3. At latitudes above approximately 35° N, winter solar zenith angles can markedly reduce the availability of UVB wavelengths required for cutaneous vitamin D synthesis, with the duration and severity of this seasonal limitation varying according to latitude, altitude, atmospheric conditions, time of day, and behavioural exposure. A KNHANES sub-analysis found that Koreans living at 37–38° N had significantly lower serum 25(OH)D and lower femoral-neck bone mineral density than those at 33–35° N, a gradient consistent with the latitude effect. These population estimates indicate that low 25(OH)D concentrations are common and are not confined to traditionally recognised high-risk subgroups, although prevalence estimates vary according to survey period, assay method, and the threshold used to define deficiency. Higher constitutive melanin content, which increases the UVB exposure required for equivalent vitamin D synthesis, may further modify the latitude effect in East-Asian and other darker-skinned populations [2,8].

### 3.2. Environmental Pollution and Atmospheric UVB Attenuation

Latitude influences the seasonal availability of UVB, while air pollution may attenuate the amount reaching ground level. Tropospheric ozone absorbs UVB photons before they reach the ground, and particulate matter—PM2.5, PM10, and the seasonal Asian dust events known in Korea as *hwangsa*—scatters and absorbs the same wavelengths. Mousavi and colleagues have argued that ambient ozone and particulate matter can meaningfully limit the UVB reaching the skin and thereby raise deficiency prevalence even in nominally sunny climates. In heavily urbanised Korea, where pollution alerts routinely encourage the population to remain indoors, this atmospheric attenuation is reinforced by a behavioural feedback loop: periods of greater atmospheric attenuation may also coincide with public-health advice or individual decisions to reduce outdoor exposure [2,15]. These pathways should be distinguished: atmospheric attenuation of UVB is a physical mechanism, behavioural avoidance during pollution episodes is a separate exposure pathway, and lower serum 25(OH)D in pollution-exposed populations remains susceptible to confounding by outdoor activity, occupation, socioeconomic status, adiposity, and other lifestyle factors. Direct human causal evidence linking ambient pollution itself to vitamin D deficiency is therefore less certain than the mechanistic rationale.

### 3.3. Behaviour: Sun Avoidance, Cosmetic Photoprotection, and High-SPF Products

Behavioural photoprotection is the third driver, and the one most amenable to intervention. The use of skin-lightening cosmetics, shade-seeking behaviour, protective clothing, and high-sun-protection-factor (SPF) products in Korea may reduce cutaneous ultraviolet-B exposure, while KNHANES analyses have linked sun-avoidant behaviour, indoor occupation, and higher educational attainment to lower serum 25-hydroxyvitamin D [25(OH)D] concentrations [7,17]. The evidence on sunscreen deserves care rather than alarm. Laboratory studies confirm that correctly and generously applied sunscreen can reduce cutaneous vitamin D synthesis, but real-world application is typically thinner, uneven, and intermittent, and field studies have generally not shown that ordinary sunscreen use produces clinically meaningful vitamin D deficiency [18,19]. This uncertainty is particularly relevant for newer high-SPF and broad-spectrum ultraviolet-filter formulations, for which real-world effects on vitamin D status may differ from laboratory photoprotection estimates and remain insufficiently characterised in long-term population studies [18]. Photoprotective behaviours other than sunscreen, especially shade-seeking, clothing coverage, and deliberate sun avoidance, may therefore be more important determinants of population vitamin D status than sunscreen use alone [7,17,19].

### 3.4. Sedentary Indoor Lifestyle and the Modern Loss of Incidental UVB Exposure

A further driver of vitamin D deficiency is the global shift from outdoor, physically active work toward indoor, sedentary, screen-based living. Historically, cutaneous vitamin D synthesis was supported not only by deliberate sun exposure, but also by incidental ultraviolet-B exposure during walking, outdoor labour, commuting, and recreation. In contemporary urban environments, this background exposure has progressively narrowed. Office work, school-based indoor activity, long commuting times, apartment living, digital entertainment, and reduced outdoor recreation all decrease the duration and body-surface area of sunlight exposure. This behavioural shift is particularly relevant because vitamin D synthesis depends on direct UVB exposure to uncovered skin, whereas time spent indoors, behind glass, or in shaded environments contributes little to cutaneous production [17,20].

Occupational data support this pattern. Systematic reviews indicate that indoor workers and shift workers have lower serum 25-hydroxyvitamin D [25(OH)D] concentrations and higher deficiency prevalence than outdoor workers. Population studies from East Asia have also linked longer sitting time and indoor occupations with lower 25(OH)D concentrations. These findings suggest that sedentary and predominantly indoor lifestyles may function both as metabolic risk factors and as markers of reduced environmental UVB exposure. Vitamin D deficiency may therefore cluster with other indoor lifestyle-related risk factors, including physical inactivity, adiposity, sarcopenia, poor sleep, cardiometabolic risk, and chronic musculoskeletal vulnerability [17,20].

These behavioural patterns interact with biological and environmental barriers. Greater skin pigmentation reduces the amount of UV radiation available for vitamin D synthesis for a given exposure, while particulate air pollution, ozone, and seasonal dust events attenuate ground-level UVB before it reaches the skin. In high-density Asian cities, including those affected by fine particulate matter and yellow dust, this creates a double barrier: less UVB reaches the urban environment, and residents respond to pollution alerts by spending more time indoors. The resulting feedback loop—pollution-driven UVB attenuation, behavioural avoidance of outdoor exposure, sedentary indoor living, and photoprotection—helps explain why vitamin D deficiency remains common even in populations that are not located at extreme latitudes [8,15].

The clinical implication is that vitamin D deficiency should not be interpreted solely as a problem of dietary insufficiency. In modern societies, it often reflects the combined influence of reduced UVB availability, limited outdoor exposure, lower cutaneous synthesis, biological modifiers, and insufficient compensatory dietary intake. This framing supports risk-based assessment and supplementation in selected individuals without encouraging unsafe sun exposure or implying that sunlight avoidance alone is the cause.

### 3.5. Ageing and Declining Cutaneous Synthesis

Ageing adds a physiological constraint to these environmental and behavioural determinants. Older skin contains less 7-dehydrocholesterol and produces less vitamin D3 for an equivalent UVB exposure than younger skin. This decline occurs in parallel with reduced outdoor activity, lower dietary intake, higher comorbidity burden, polypharmacy, reduced renal activation of vitamin D in some patients, and increased risk of sarcopenia and falls. As a result, older adults can become deficient even when ambient sunlight is available and dietary intake appears adequate [16].

## 4. Musculoskeletal Consequences

The musculoskeletal system provides the clearest biological rationale and the most clinically established indication for recognising and correcting vitamin D deficiency. The evidence is strongest for osteomalacia, calcium–phosphate homeostasis, skeletal mineralisation, muscle function, and optimisation of osteoporosis care in deficient or high-risk individuals. Importantly, these effects are not limited to bone: vitamin D-related pathways also intersect with skeletal muscle biology, neuromuscular function, inflammatory signalling, and pain vulnerability. However, recent fracture-prevention trials and meta-analyses show that routine supplementation of broadly unselected community-dwelling adults should not be expected to prevent fractures or falls as a stand-alone intervention [1,6,16].

### 4.1. Mechanistic Links Among Vitamin D, Muscle, Bone, and Pain

The musculoskeletal relevance of vitamin D extends beyond bone mineralisation. Severe deficiency impairs calcium absorption and osteoid mineralisation, producing osteomalacia, diffuse bone pain, proximal muscle weakness, and gait disturbance [1]. However, vitamin D also has direct and indirect roles in skeletal muscle and pain-related pathways. Vitamin D receptor-related signalling has been identified in skeletal muscle, and the vitamin D endocrine system has been linked to muscle-cell differentiation, intracellular calcium handling, genomic activity, mitochondrial function, proteolysis, cellular senescence, adiposity, and muscle protein turnover [21,22,23]. These mechanisms provide biological plausibility for the association between low 25(OH)D, reduced muscle strength, sarcopenia, impaired balance, and falls [24,25].

Vitamin D deficiency may also contribute to musculoskeletal pain through mechanisms that are not purely skeletal. Osteomalacic pain reflects defective mineralisation, but chronic musculoskeletal pain in deficient individuals may also involve low-grade inflammation, altered neuromuscular function, reduced physical activity, sleep disruption, and increased pain sensitivity. Systematic reviews have reported associations between lower 25(OH)D concentrations and pain conditions, including arthritis, muscle pain, and chronic widespread pain, although the evidence is heterogeneous and does not establish vitamin D as an analgesic treatment [26,27]. More recent large-scale data also suggest that severe vitamin D deficiency may be more relevant to chronic widespread pain than to regional musculoskeletal pain [28]. The clinically useful interpretation is therefore a vulnerability model: vitamin D deficiency may contribute to greater musculoskeletal pain and functional vulnerability in susceptible patients, particularly when combined with sarcopenia, poor sleep, obesity, low activity, osteomalacia, or inflammatory comorbidity. Correction of documented deficiency should be viewed as one component of multimodal musculoskeletal care, not as a stand-alone pain intervention.

### 4.2. Osteoporosis and Osteopenia

Osteoporosis and osteopenia are highly prevalent among older Korean adults. KNHANES data from 2008–2011 indicated osteoporosis in approximately 37% of women and 7.5% of men aged 50 years and older, with osteopenia in a further 47–49% of each sex; over the subsequent decade, age-standardised osteoporosis prevalence increased in both sexes. Vitamin D is necessary but not sufficient for bone health: inadequate 25(OH)D reduces intestinal calcium absorption, promotes compensatory parathyroid hormone elevation, and may accelerate cortical bone loss through secondary hyperparathyroidism. Correction of deficiency does not by itself treat established osteoporosis, but adequate vitamin D status is an important component of osteoporosis management and should be optimised when antiresorptive or anabolic therapy is used [1,16,29,30]. Recent Korean genetic data also suggest that inherited variation may modify bone-related phenotypes and vitamin D-associated biochemical characteristics, although exploratory genetic associations should not be interpreted as deterministic population-level risk or as justification for ancestry-specific supplementation thresholds [31].

### 4.3. Osteomalacia and the Threat to Peak Bone Mass

Two distinct problems arise at the extremes of adulthood. In older adults, prolonged severe vitamin D deficiency can produce osteomalacia, a disorder of defective osteoid mineralisation that may present with diffuse bone pain, bone tenderness, proximal muscle weakness, gait disturbance, and increased fracture susceptibility [1,32]. Because these manifestations are often nonspecific, osteomalacia may be confused with fibromyalgia, polymyalgia rheumatica, polymyositis, inflammatory back pain, spondyloarthropathy, or other chronic musculoskeletal pain syndromes if vitamin D status and mineral metabolism are not assessed [33]. Recognising osteomalacic pain as a potentially treatable consequence of severe deficiency is therefore clinically important, particularly in older adults with diffuse pain, proximal weakness, low activity, or unexplained functional decline.

In young adults, the problem is different but no less relevant. KNHANES data show that Koreans in their twenties have among the lowest 25-hydroxyvitamin D [25(OH)D] concentrations and the highest deficiency prevalence of any age group, with deficiency reported in approximately 65% of men and 80% of women [7]. This period is clinically important because peak bone mass is largely accrued by the end of the second decade and continues to consolidate into early adulthood; the skeletal reserve achieved during this period is a major determinant of lifelong bone strength and later osteoporosis risk [34]. Vitamin D deficiency during this window should not be interpreted as an immediate fracture-risk equivalent, but it may represent a long-term skeletal vulnerability, particularly when combined with low physical activity, low calcium intake, indoor living, suboptimal dietary patterns, or other nutritional and endocrine risk factors.

### 4.4. Hip Fracture: A High-Mortality Endpoint

The clinical stakes of the deficiency–bone axis are clearest at the hip. One-year mortality after hip fracture in patients aged 65 and over is on the order of 15–20% in systematic reviews, with some recent series reporting figures approaching one third; in United States data the one-year rate is approximately 21% in those over 60. Excess mortality is not confined to the first year—annual mortality remains elevated in subsequent years, and long-term survival is markedly worse than in age-matched controls. Against a demographic backdrop in which the global number of hip fractures is projected to reach roughly 4.5 million per year by 2050, interventions that meaningfully reduce fracture risk would have substantial population-level implications. Vitamin D correction remains clinically relevant in patients with documented deficiency, osteomalacia, institutionalisation, or concurrent osteoporosis treatment, but it should not be interpreted as a stand-alone hip-fracture prevention strategy [35,36].

### 4.5. Sarcopenia and the Falls–Fracture Pathway

Sarcopenia—present in an estimated 5–13% of people in their sixties and in a substantially higher proportion of those over eighty—is an age-related condition and an important contributor to falls and fracture risk. A more appropriate clinical framework is therefore an interacting vulnerability pathway in which vitamin D deficiency may coexist with sarcopenia, impaired balance, frailty, falls, and fracture susceptibility. These factors are potentially modifiable through combined nutritional, exercise-based, fall-prevention, and osteoporosis-specific interventions, with correction of documented vitamin D deficiency forming one component of care [24,25].

### 4.6. Calcium, Vitamin D, and Fracture Prevention: Boundary Conditions from Recent Evidence

The skeletal importance of vitamin D should not be interpreted as evidence that routine supplementation prevents fractures or falls in all adults. A 2026 BMJ systematic review and meta-analysis evaluated calcium, vitamin D, and combined calcium–vitamin D supplementation for fracture and fall prevention in adults who were not receiving pharmacological osteoporosis treatment, most of whom were community dwelling and not at high fracture risk. Across 69 randomised trials involving 153,902 participants, vitamin D alone had essentially no effect on any fracture (risk ratio 1.00, 95% confidence interval 0.95–1.06; high-certainty evidence), while combined calcium–vitamin D supplementation produced a small relative reduction (risk ratio 0.91, 0.84–0.99; high-certainty evidence). The investigators judged the corresponding absolute effects to remain below their prespecified threshold for clinical importance. Thus, statistical significance for the combined regimen should not be equated with a clinically meaningful population benefit. Evidence was limited for high-risk patients and those requiring residential care, and this absence of evidence should not be interpreted as proof of benefit in those groups. These findings delineate the boundary of routine supplementation rather than negate the biological requirement for vitamin D in calcium absorption, osteoid mineralisation, muscle function, or the management of established deficiency [6,16].

## 5. Cardiovascular Disease and Atherosclerosis

Cardiovascular disease is the domain in which vitamin D requires the most cautious interpretation. The biological rationale is plausible because vitamin D signalling intersects with endothelial function, vascular inflammation, renin–angiotensin–aldosterone system activity, and vascular calcification pathways [37,38]. Observational associations between low 25(OH)D and cardiovascular risk are frequently reported, but randomised supplementation trials and updated meta-analyses have not demonstrated a clear cardiovascular primary-prevention benefit [4,39]. This section therefore frames low vitamin D status primarily as a vascular risk marker associated with atherosclerotic dysfunction, rather than as an established therapeutic target for cardiovascular disease prevention.

### 5.1. Biological Plausibility: Vascular Inflammation, Endothelial Dysfunction, and Calcification

Vitamin D signalling intersects with several pathways central to atherogenesis. The vitamin D receptor is expressed in vascular endothelial cells, vascular smooth muscle cells, cardiomyocytes, and immune cells involved in plaque biology. Through these sites, 1,25-dihydroxyvitamin D may influence endothelial nitric oxide bioavailability, oxidative stress, renin–angiotensin–aldosterone system activity, macrophage activation, vascular smooth muscle cell phenotype, lipid handling, and inflammatory cytokine signalling. These mechanisms are directly relevant to endothelial dysfunction, arterial stiffness, vascular calcification, and plaque progression [37,38].

The relationship between vitamin D and vascular calcification is particularly nuanced. Experimental and observational evidence suggests that vitamin D signalling may interact with inflammation-related vascular mineralisation. Severe deficiency may coexist with pro-inflammatory and adverse cardiometabolic conditions, whereas excessive vitamin D exposure, particularly in the setting of calcium–phosphate imbalance or chronic kidney disease, may aggravate vascular calcification. Vitamin D should therefore not be conceptualised as uniformly anti-atherosclerotic; rather, its vascular effects are likely to be dose-, context-, and comorbidity-dependent [38].

### 5.2. Observational Associations: Consistent, but Vulnerable to Confounding

Population-based studies and meta-analyses have repeatedly reported associations between low serum 25-hydroxyvitamin D [25(OH)D] and hypertension, arterial stiffness, coronary artery disease, vascular calcification, cardiovascular events, and cardiovascular mortality. These findings support the concept that vitamin D deficiency often accompanies an adverse cardiometabolic phenotype. However, they do not establish causality. Low 25(OH)D may be a mediator, a marker, or both. Individuals with low vitamin D status frequently have less outdoor activity, higher adiposity, older age, poorer diet quality, diabetes, chronic kidney disease, systemic inflammation, or reduced physical function, all of which independently increase cardiovascular risk [38,39].

This distinction is important for the present review. The observational signal remains clinically relevant because it may identify patients with broader nutritional, metabolic, or functional vulnerability. At the same time, it should not be over-interpreted as proof that vitamin D supplementation prevents atherosclerosis or cardiovascular events.

### 5.3. Randomised Trial Evidence: Neutral for Cardiovascular Primary Prevention

The randomised evidence is considerably less supportive than the observational literature. In the VITAL trial, daily vitamin D3 supplementation at 2000 IU did not reduce the primary composite cardiovascular endpoint of myocardial infarction, stroke, or cardiovascular death, nor did it reduce individual cardiovascular endpoints. Subsequent meta-analyses of randomised trials have similarly failed to show convincing reductions in major adverse cardiovascular events, cardiovascular mortality, or all-cause mortality [4,39,40].

These neutral findings are clinically important, but they should be interpreted according to trial design. Most large trials enrolled broad, generally healthy populations without selecting for severe vitamin D deficiency, did not individualise dosing to baseline 25(OH)D or body mass index, and often tested fixed-dose supplementation rather than target-based repletion. Therefore, these trials primarily answer the question of whether routine supplementation benefits an unselected population; they do not directly establish whether correcting documented deficiency in selected high-risk cardiovascular patients improves vascular outcomes [4,5].

### 5.4. Genetic Evidence and Retracted Non-Linear Mendelian Randomisation

Mendelian randomisation studies have attempted to clarify whether low vitamin D status is causally related to cardiovascular disease. Conventional linear Mendelian randomisation has not provided strong evidence for a causal cardiovascular effect across the usual population range of serum 25-hydroxyvitamin D [25(OH)D] concentrations. Earlier enthusiasm was partly driven by a non-linear Mendelian randomisation analysis suggesting that cardiovascular benefit might be concentrated in the deficient range [41]. However, this line of evidence should now be treated with caution. The key European Heart Journal report has since been formally retracted, and subsequent methodological critique argued that non-linear Mendelian randomisation publications on vitamin D may generate spurious findings when model assumptions are violated [42,43]. Accordingly, this review does not use the retracted non-linear Mendelian randomisation evidence as support for a cardiovascular benefit of vitamin D supplementation. The retracted report is retained only briefly for methodological context because it previously influenced discussion of a possible non-linear deficiency threshold; it is not used as supporting evidence for any clinical conclusion.

### 5.5. Clinical Interpretation

The most defensible interpretation is qualified. Vitamin D deficiency is biologically and epidemiologically linked to vascular dysfunction and atherosclerotic cardiovascular disease, but vitamin D supplementation should not be presented as an established strategy for cardiovascular primary prevention. In clinical practice, low 25(OH)D may be considered one component of a broader cardiometabolic risk phenotype, particularly in older adults and in patients with obesity, diabetes, chronic kidney disease, limited sun exposure, low physical activity, or coexisting musculoskeletal disease. Correcting documented deficiency remains reasonable for skeletal, muscular, and general nutritional indications, but any cardiovascular benefit remains unproven, and vitamin D treatment should not replace established cardiovascular interventions such as blood-pressure control, lipid lowering, smoking cessation, diabetes management, physical activity, weight reduction, and antiplatelet therapy when indicated [4,39].

Emerging target-based trials in secondary prevention populations may refine this conclusion, especially if they enrol vitamin-D-deficient patients and adjust dosing to achieved 25(OH)D concentrations. Until such evidence is published and replicated, the cardiovascular role of vitamin D should be framed as plausible and clinically relevant, but not proven.

## 6. Immunological and Oncological Implications

### 6.1. Vitamin D as an Immunomodulator

Many immune-cell populations—including T lymphocytes, B lymphocytes, macrophages, dendritic cells, and natural killer cells—express the vitamin D receptor or participate in local vitamin D metabolism, providing a mechanistic basis for immunomodulatory effects. The active metabolite 1,25-dihydroxyvitamin D3 can support innate immune responses through the induction of antimicrobial peptides, including cathelicidin and β-defensin. In adaptive immunity, vitamin D signalling has been associated with modulation of T-helper-cell responses, regulatory T-cell activity, nuclear factor-κB signalling, and inflammatory cytokine production. The overall effect is better characterised as immunoregulatory than simply immunostimulatory—a distinction that is relevant when considering infection, inflammation, and cancer [44,45].

### 6.2. Cancer: Associations, Mechanisms and the Trial Reality

The oncological evidence should be interpreted by separating mechanistic plausibility and observational associations from randomised trial outcomes. Mechanistically, 1,25-dihydroxyvitamin D3 has been reported to influence cell-cycle regulation, apoptosis, angiogenesis, and immune-cell activity in experimental models; possible interactions with antibody-dependent cellular cytotoxicity have also been described. Epidemiologically, a comprehensive meta-meta-analysis reported an association between higher serum 25(OH)D concentrations and an approximately 20% lower cancer risk. However, randomised evidence is more conservative: in the large primary-prevention trials, vitamin D supplementation did not reduce cancer incidence [4,45,46,47].

A potentially more clinically relevant signal has been observed for cancer mortality rather than cancer incidence. An individual-patient-data meta-analysis of fourteen randomised trials involving 104,727 participants found a statistically non-significant 6% reduction in cancer mortality overall (relative risk 0.94, 95% confidence interval 0.86–1.02). When the analysis was restricted to trials using daily dosing, cancer mortality was reduced by 12% (relative risk 0.88, 0.78–0.98), whereas bolus dosing showed no benefit (relative risk 1.07, 0.91–1.24; *p* for interaction = 0.042). Subgroup analyses suggested greater benefit in adults aged 70 years and older and when supplementation was initiated before a cancer diagnosis. These data do not establish vitamin D as a cancer-preventive therapy, but they support the broader interpretation that baseline status, dosing schedule, endpoint selection, and population risk influence whether clinical effects are detectable [48]. Importantly, the apparent difference between daily and bolus regimens arose from subgroup comparisons across trials rather than direct randomisation to dosing frequency and should therefore remain hypothesis-generating rather than evidence that daily dosing is superior for cancer outcomes.

### 6.3. Respiratory Infection: Evolving Evidence and Implications for Dosing

Respiratory infection illustrates both the biological plausibility of vitamin D-mediated immune effects and the need to update conclusions as trial evidence evolves. Earlier individual-participant-data meta-analyses suggested a small protective effect of vitamin D supplementation against acute respiratory infections, with greater benefit in daily or weekly regimens and in participants with lower baseline 25(OH)D. However, the 2025 updated aggregate-data meta-analysis, which incorporated six additional trials and more than 61,000 participants, no longer found a statistically significant reduction in overall acute respiratory infection risk. This newer evidence argues against presenting vitamin D as a general respiratory-infection prevention strategy in unselected populations. Nevertheless, it remains relevant to the dosing discussion because the 2024 Endocrine Society guideline generally favours daily lower-dose vitamin D over intermittent high-dose regimens in adults for whom supplementation is indicated [49,50].

The COVID-19 literature provides an example of the same evidence hierarchy. Observational studies associated low 25(OH)D with greater disease severity, but these findings were vulnerable to confounding by age, obesity, frailty, comorbidity, reduced outdoor activity, and illness severity. Interventional findings have been heterogeneous: small calcifediol studies and selected meta-analyses reported favourable signals, whereas a large trial of a single high-dose vitamin D3 bolus and the CORONAVIT test-and-treat trial did not demonstrate major clinical or preventive benefit. Overall, current evidence does not support vitamin D as a stand-alone antiviral or acute COVID-19 treatment; correction of documented deficiency should instead follow established nutritional or skeletal indications [51,52,53,54].

## 7. Neuropsychiatric Dimensions

The neuropsychiatric associations are supported predominantly by observational and mechanistic evidence and are therefore presented cautiously throughout. They remain clinically relevant because mood, anxiety, sleep disturbance, and chronic pain frequently coexist with nutritional, inflammatory, and behavioural risk factors associated with low vitamin D status [28,55,56].

### 7.1. Depression

Cross-sectional and longitudinal studies consistently report lower serum 25-hydroxyvitamin D [25(OH)D] concentrations in people with depression than in controls, but these associations remain vulnerable to confounding by outdoor activity, physical illness, adiposity, sleep disturbance, inflammation, and socioeconomic factors [55]. Updated randomised-trial meta-analytic evidence suggests that any improvement in depressive symptoms may be more likely in clinically depressed or vitamin-D-deficient subgroups than in broadly unselected or vitamin-D-replete populations [57]. Mechanistically, the vitamin D receptor and the activating enzyme 1α-hydroxylase are distributed in brain regions relevant to mood regulation, including the prefrontal cortex, hippocampus, amygdala, and hypothalamus, and vitamin D signalling has been linked to serotonergic, inflammatory, and neuroimmune pathways [55,56]. A cautious clinical interpretation is that correction of documented vitamin D deficiency may be considered as an adjunctive nutritional measure in patients with depression, but supplementation should not be framed as an antidepressant treatment in vitamin-D-replete individuals [55,57].

### 7.2. Anxiety

Anxiety has been less extensively studied than depression in the vitamin D literature, and the available evidence supports a cautious association rather than a treatment claim. Meta-analytic evidence suggests that vitamin D supplementation has been associated with modest improvements in negative emotional symptoms in some selected populations, particularly among individuals with low baseline 25(OH)D or clinically significant depressive symptoms, but the findings are heterogeneous and anxiety-specific effects remain less consistent [58]. Cross-sectional data have also linked higher dietary vitamin D intake with lower odds of anxiety, although residual confounding by outdoor activity, physical health, diet quality, and socioeconomic factors remains difficult to exclude [59]. Mechanistically, this association is biologically plausible because vitamin D participates in immune regulation, inflammatory signalling, and neurotransmitter-related pathways, all of which have been implicated in anxiety-related pathophysiology [55]. However, vitamin D supplementation should not be framed as an anxiolytic intervention. Its more defensible role is as part of a broader nutritional and metabolic assessment in patients with anxiety symptoms who are also deficient or otherwise at high risk of deficiency.

### 7.3. Sleep Disturbance, Pain Amplification, and Chronic Pain Vulnerability

Sleep disturbance deserves separate consideration because it links the neuropsychiatric and musculoskeletal consequences of vitamin D deficiency. Observational evidence and meta-analytic data have associated low 25-hydroxyvitamin D [25(OH)D] status with poor sleep quality, shorter sleep duration, daytime sleepiness, and sleep disorders [60]. Intervention-focused systematic review evidence has reported possible improvements in sleep quality in selected populations, although effects on sleep duration and specific sleep disorders remain uncertain [61]. The mechanistic basis is plausible but not definitive. Vitamin D receptors and vitamin-D-metabolising enzymes are present in brain regions involved in sleep regulation, and vitamin D may influence circadian biology through inflammatory modulation, serotonergic signalling, and downstream effects on melatonin-related pathways. A proposed mechanistic model links vitamin D signalling with serotonergic and melatonin-related pathways, although this framework remains hypothetical and has not been established as a clinical mechanism [56].

The clinical importance of sleep is not limited to mood. Sleep disturbance is a recognised amplifier of pain sensitivity and may contribute to the transition from episodic musculoskeletal pain to persistent or widespread pain. Experimental, prospective, and clinical literature suggests that insufficient sleep can lower pain thresholds, increase central pain sensitivity, impair descending inhibitory control, and reinforce chronic pain through reciprocal sleep–pain interactions [62]. This is particularly relevant to clinical nutrition and musculoskeletal practice because vitamin D deficiency may coexist with osteomalacic pain, sarcopenia, low physical activity, depression, and sleep disruption in the same patient. More recent large-scale data also suggest that severe vitamin D deficiency may be more relevant to chronic widespread pain than to regional musculoskeletal pain [28].

The evidence should therefore be interpreted as a vulnerability model rather than a simple causal chain. Vitamin D deficiency does not independently explain insomnia or chronic pain, and supplementation should not be presented as a primary treatment for either condition in vitamin-D-replete individuals. However, correction of documented deficiency may be reasonable as part of multimodal care in patients with chronic widespread pain, fatigue, depressive symptoms, or poor sleep.

## 8. Reconciling Observational Associations and Neutral Supplementation Trials

**Figure 2:**
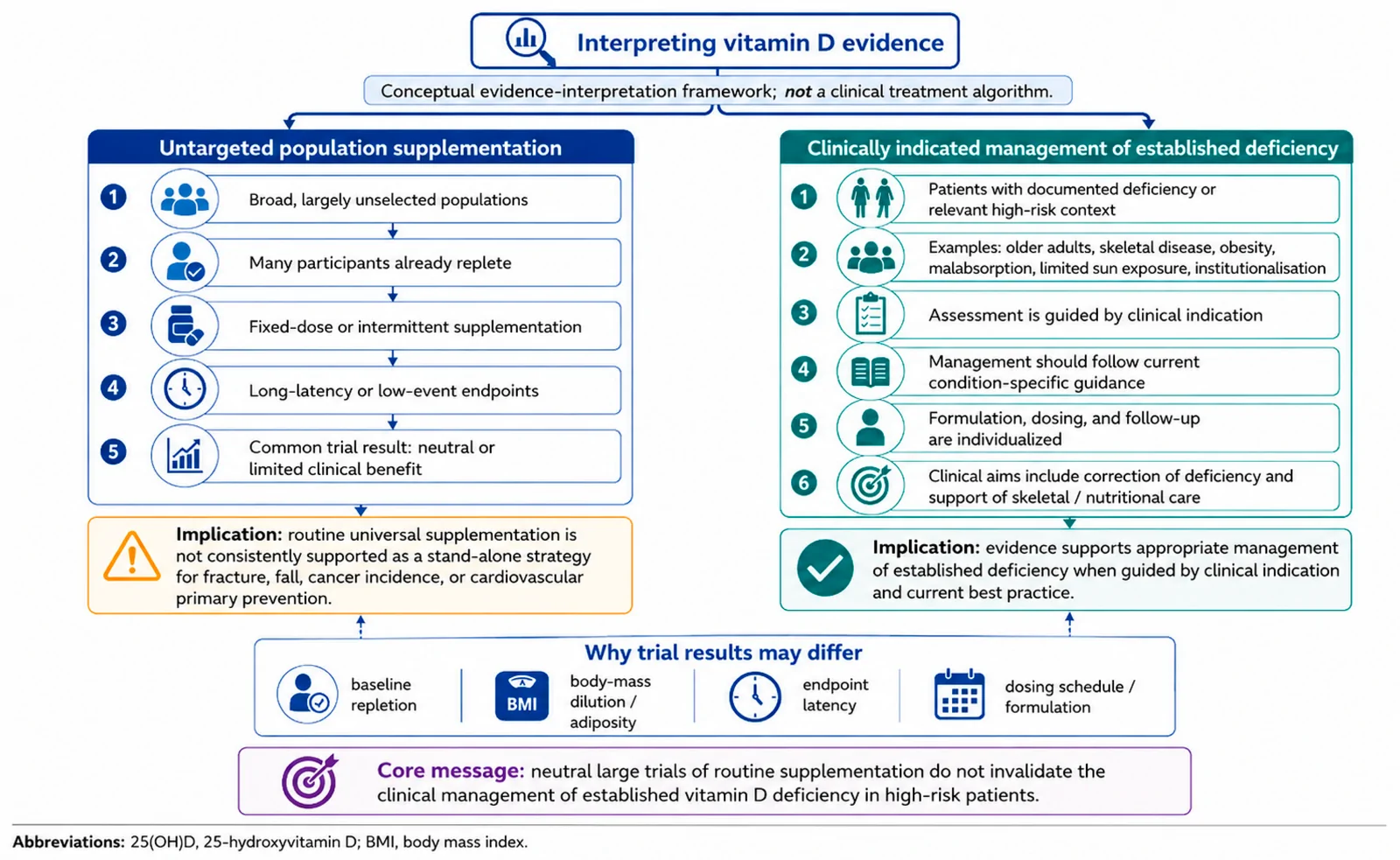
Interpreting neutral supplementation trials through a targeted-repletion framework. The figure contrasts routine supplementation in broad, largely unselected populations with clinically indicated management of documented deficiency or a separate established indication for assessment. This is a conceptual evidence-interpretation framework, not a treatment pathway or dosing algorithm. Baseline status, adiposity, endpoint latency, formulation, and dosing schedule are shown as factors that may influence trial findings. Generated using ChatGPT Images 2.0 (OpenAI, 2026) from author-specified scientific content; the final figure was critically reviewed and approved by all authors for scientific accuracy.

The major interpretive challenge in the vitamin D literature is the discordance between mechanistic and observational findings and largely neutral large-scale randomised supplementation trials. Trials such as VITAL, D-Health, and ViDA, together with recent fracture-prevention meta-analyses, primarily evaluated fixed-dose or routine supplementation in broad populations, many of whom were not selected for documented vitamin D deficiency, low bone mass, high fracture risk, or a specific deficiency-related clinical syndrome. These studies provide important boundary conditions for claims about universal supplementation. However, they do not fully answer the narrower clinical question of whether correcting genuine deficiency in selected high-risk individuals improves skeletal, muscular, nutritional, or other clinically relevant outcomes. Four design features may contribute to this distinction: baseline repletion, body-mass dilution, endpoint latency, and dosing schedule. This distinction between untargeted population supplementation and clinically indicated management of established deficiency is summarised in Figure 2 [4,6,63,64].

### 8.1. Baseline Repletion and the Ceiling Effect

A trial is best positioned to test the clinical value of deficiency correction when it enrols participants with documented deficiency or prespecifies analyses by baseline 25(OH)D status. VITAL was not primarily designed around this question: among participants with baseline 25(OH)D measurements, the median concentration was approximately 31 ng/mL (78 nmol/L), and only a minority were vitamin-D-deficient. Supplementation in largely replete populations therefore mainly tests the effect of additional vitamin D exposure above conventional adequacy, rather than the correction of established deficiency; a null result in the former context should not be interpreted as definitive evidence against the latter [4]. D-Health was similarly designed as a population-level supplementation trial in older Australian adults recruited without baseline vitamin D screening, and baseline 25(OH)D was not measured in all participants. These trials provide important evidence against routine untargeted supplementation in broad populations, but they do not directly test condition-specific management of documented deficiency [63].

### 8.2. Body-Mass Dilution

Adiposity is an important determinant of achieved 25(OH)D concentration because vitamin D is fat-soluble and may be sequestered or volumetrically diluted in larger body compartments; consequently, a fixed dose generally produces a smaller rise in serum 25(OH)D in individuals with obesity than in those with normal body weight [65,66]. This pharmacokinetic issue is relevant to trial interpretation. In VITAL subgroup analyses, participants with a normal body mass index (<25 kg/m^2^) showed a lower incidence of total cancer with supplementation, whereas overweight and obese participants did not show a comparable association [40]. These findings were derived from subgroup analyses and should be regarded as hypothesis-generating rather than evidence for BMI-specific vitamin D treatment. Nevertheless, they illustrate how fixed-dose supplementation may produce heterogeneous biochemical and clinical responses across body-size groups. Inter-individual response may also reflect biological factors not captured by serum 25(OH)D alone. Recent orthopedic data suggest that VDR rs2228570 (FokI) genotype may be associated with differences in inflammatory and patient-reported outcomes, supporting further study of genotype–phenotype variability without establishing genotype-guided treatment at present [67].

### 8.3. Endpoint and Latency

Cancer incidence and cancer mortality are biologically and temporally distinct endpoints, and vitamin D trials have shown more consistent signals for mortality than for incidence. In VITAL, vitamin D supplementation did not reduce total invasive cancer incidence, but secondary analyses excluding the first one to two years of follow-up suggested a reduction in total cancer mortality, a finding consistent with the possibility that vitamin D may be more relevant to tumour progression or survival than to cancer initiation [4,40]. The individual-patient-data meta-analysis by Kuznia and colleagues made a similar point at scale: the overall 6% reduction in cancer mortality across all trials was not statistically significant, but daily vitamin D3 supplementation was associated with a 12% reduction in cancer mortality, whereas bolus dosing showed no benefit [48]. These findings do not establish vitamin D as a cancer therapy or cancer-preventive intervention, but they illustrate why endpoint selection, latency, dosing schedule, and population risk are central to interpreting neutral supplementation trials.

### 8.4. Dosing Schedule

The fourth feature links trial interpretation to dosing strategy. Some clinical signals, particularly in cancer-mortality analyses, have been reported more consistently with daily lower-dose regimens than with large intermittent bolus regimens [48]. Earlier individual-participant-data meta-analyses of acute respiratory infection also suggested greater benefit with daily or weekly dosing than with large intermittent bolus regimens, although updated aggregate-data evidence has made the overall respiratory-infection signal less certain [49,50]. Large infrequent doses can produce wider fluctuations in 25(OH)D and may alter vitamin D metabolism in ways that differ from physiological daily exposure. This does not prove that daily dosing improves all clinical outcomes, but it is consistent with guideline preferences for daily lower-dose regimens when supplementation is indicated [5].

The same distinction applies to skeletal outcomes. As discussed in Section 4.6, a recent fracture- and fall-prevention meta-analysis found little to no clinically meaningful benefit from routine calcium, vitamin D, or combined supplementation in broadly unselected adults not receiving pharmacological osteoporosis treatment [6]. This finding should not be interpreted as evidence that documented vitamin D deficiency is clinically irrelevant in patients with osteomalacia, malabsorption, institutionalisation, high skeletal risk, or osteoporosis treatment requirements. Instead, it reinforces the central distinction of this review: untargeted supplementation and targeted deficiency correction are not the same intervention.

Practical pharmacokinetic comparisons of daily-equivalent weekly or monthly replacement and the safety concerns associated with very large intermittent boluses are discussed in Section 10, where dosing and route are considered in their clinical management context.

## 9. Interpreting Serum 25(OH)D Targets

### 9.1. Current Guideline Landscape

Any discussion of serum 25(OH)D concentrations above conventional skeletal adequacy thresholds must be interpreted within the current guideline landscape. The Institute of Medicine 2011 dietary reference framework considered serum 25(OH)D concentrations of approximately 20 ng/mL (50 nmol/L) or higher adequate for skeletal health in most individuals [68]. The 2011 Endocrine Society guideline historically used a higher threshold of 30 ng/mL (75 nmol/L) and provided explicit deficiency-treatment regimens [69]. However, the official correction to the 2024 Endocrine Society guideline clarifies that the 2024 guideline replaces the previous 2011 guideline, specifically to avoid the impression that the earlier guideline remains current [70]. The 2024 document primarily addresses vitamin D use for disease prevention in people without established indications for testing or treatment and does not endorse a universal serum target of 30 ng/mL (75 nmol/L) or fixed categories of sufficiency and insufficiency for generally healthy populations [5]. Contemporary consensus statements likewise emphasise that interpretation of 25(OH)D depends on the clinical question, population risk, assay context, and intended outcome [71]. Historical dosing regimens may still be discussed for context, but they are identified as historical rather than current Endocrine Society therapeutic standards. The principal distinctions among current, historical, and expert frameworks are summarized in Table 1.

### 9.2. Interpreting Proposed Higher Serum 25(OH)D Ranges

Proposed serum 25-hydroxyvitamin D [25(OH)D] ranges of 50–80 ng/mL should not be interpreted as population-wide screening thresholds, universal supplementation goals, or established therapeutic targets. Such ranges have been proposed by selected investigators for monitored use in individuals with documented deficiency or specific clinical vulnerabilities, but direct randomised evidence demonstrating additional clinical benefit over conventional skeletal adequacy thresholds remains limited [72]. This distinction is important in the context of the 2024 Endocrine Society guideline, which addresses vitamin D use for disease prevention in generally healthy populations and advises against routine 25(OH)D screening and empiric high-dose supplementation in individuals without established indications for testing or treatment [5]. Whether selected patients with severe deficiency, malabsorption, obesity, high skeletal risk, limited ultraviolet exposure, or other vulnerability phenotypes benefit from achieving concentrations above conventional adequacy thresholds remains an unresolved clinical question rather than an established treatment principle.

Safety considerations should be addressed separately from assumptions regarding clinical benefit. Vitamin D toxicity is not defined by serum 25(OH)D concentration alone, but by a biochemical and clinical syndrome characterised by hypercalcaemia, hypercalciuria, suppressed parathyroid hormone, nephrocalcinosis, and renal impairment, usually following excessive vitamin D exposure [73,74]. Case reports indicate that overt toxicity is most commonly observed at markedly elevated 25(OH)D concentrations, often above 150 ng/mL, particularly when accompanied by hypercalcaemia and excessive dosing [74]. Nevertheless, the absence of overt toxicity at lower concentrations should not be interpreted as evidence of additional therapeutic benefit. Consistent with ESPEN guidance on micronutrient management, when higher-dose vitamin D treatment is used for a clinically justified indication, monitoring may include serum calcium, renal function, vitamin D and calcium intake, symptoms of hypercalcaemia, and urinary calcium when clinically indicated [75].

### 9.3. A Contextual Framework for Serum 25(OH)D Interpretation

Table 2 provides a deliberately simplified contextual framework rather than finely divided therapeutic target bands. It is not a treatment algorithm. Concentrations above conventional skeletal adequacy thresholds are grouped together because current evidence does not validate incremental higher serum targets [5,68,69,71,76].

## 10. Risk-Stratified Vitamin D Management, Dosing, and Route

Clinical management should begin by determining whether there is an established indication for assessment or treatment. Once an indication is established, dosing, formulation, route, and follow-up should be individualized according to the underlying condition, adherence, absorption, safety considerations, and applicable contemporary guidance [5,77,78]. In practice, at-risk patients should be tested when the result is expected to alter management; if deficiency is documented, treatment should use an evidence-supported repletion regimen followed by maintenance therapy, with daily or daily-equivalent weekly or modest monthly oral schedules selected according to clinical context, adherence, and contemporary guidance, and with selective biochemical reassessment. Current maintenance-oriented frameworks generally span approximately 800–2000 IU/day depending on clinical purpose and population, whereas time-limited higher-dose repletion is reserved for selected patients with documented deficiency and should follow condition-specific or regional guidance.

### 10.1. Who Should Be Assessed or Treated?

The first management decision is whether an individual is generally healthy or has an established clinical indication for assessment. Routine 25(OH)D screening is not recommended solely because of age below 75 years, obesity, dark complexion, or residence in a region with common deficiency. Testing is more defensible when the result is expected to alter management, including suspected osteomalacia or rickets, hypocalcaemia, unexplained proximal muscle weakness, malabsorption or bariatric surgery, fragility fracture or osteoporosis treatment, severe dietary restriction, institutionalisation or prolonged lack of ultraviolet exposure, chronic use of medications that accelerate vitamin D metabolism, or recurrent deficiency despite supplementation [5,16,68,69,79].

Before high-dose repletion, clinicians should review total vitamin D and calcium exposure and identify conditions requiring modified treatment or specialist oversight, including hypercalcaemia, nephrolithiasis, advanced chronic kidney disease, primary hyperparathyroidism, granulomatous disease, lymphoma, or disorders of vitamin D metabolism. Baseline assessment may include serum 25(OH)D, calcium, creatinine or estimated glomerular filtration rate, phosphate, alkaline phosphatase, and parathyroid hormone when severe deficiency or osteomalacia is suspected. A practical risk-stratified synthesis of these considerations is provided at the end of this section.

### 10.2. Practical Dosing Principles

Practical dosing should be individualized according to baseline 25(OH)D concentration, body size, dietary intake, malabsorption risk, adherence, comorbidity, and the intended clinical objective. For osteoporosis care and skeletal adequacy, the Korean position statement recommends 800–1000 IU/day in many adults, while higher doses may be required for the correction of documented deficiency or in patients with obesity or malabsorption [16,77,80]. Because serum responses vary substantially, treatment should be guided by clinical indication and biochemical reassessment rather than by a universal weight-based regimen or a fixed dose intended to achieve concentrations above conventional adequacy thresholds. Intramuscular administration remains an option in selected patients with malabsorption or persistent non-adherence.

The Korean recommendation of 800–1000 IU/day should therefore be interpreted as a maintenance-oriented skeletal-health framework rather than as a universal treatment dose. International documents differ because they address different populations and objectives. The IOM/NASEM dietary reference framework uses 600 IU/day for adults aged 19–70 years and 800 IU/day for those older than 70 years, while osteoporosis-oriented guidance commonly uses 800–1000 IU/day. The 2011 Endocrine Society guideline historically described 6000 IU/day or 50,000 IU/week of vitamin D2 or D3 for 8 weeks followed by maintenance, but this guideline has been formally replaced and the regimen is retained here only as historical context. The Central and Eastern European expert consensus describes 800–2000 IU/day for maintenance and permits 6000 IU/day for 4–12 weeks when rapid correction is clinically indicated. These figures should be interpreted according to source status and clinical context rather than combined into a single universal algorithm [5,16,68,69,79]. These differing frameworks are summarized in Table 3.

### 10.3. Route and Dosing Frequency

Intramuscular cholecalciferol has a different pharmacokinetic profile from repeated oral supplementation and may be less suitable when rapid dose adjustment or close biochemical titration is required. After intramuscular administration, cholecalciferol behaves as a depot preparation, with delayed and variable systemic release related to its lipophilicity, local tissue retention, and subsequent redistribution into adipose stores. This profile may be useful when adherence to oral therapy is poor or intestinal absorption is impaired, although the timing and magnitude of the serum response may be less predictable than with repeated oral dosing.

Available pharmacokinetic data support this distinction. Wylon et al. reported that a single 100,000 IU intramuscular dose increased mean serum 25-hydroxyvitamin D [25(OH)D] to 70.9 nmol/L, approximately 28.4 ng/mL, at 4 weeks, but approximately half of treated participants still remained vitamin D deficient after a single injection [78]. In the same study, oral cholecalciferol produced a more rapid increase in 25(OH)D, although levels declined more quickly after discontinuation. Thus, intramuscular administration offers an adherence advantage but provides less immediate control over the timing and magnitude of the serum response.

Intermittent high-dose bolus regimens raise a related but distinct concern. Large oral or parenteral boluses may transiently increase circulating 25(OH)D but can produce wider peak–trough variation than repeated daily dosing, which may be less compatible with individualised biochemical monitoring [77]. For patients in whom a higher monitored 25(OH)D range is being considered, repeated lower-dose oral administration with biochemical reassessment is generally more compatible with dose titration, safety monitoring, and sustained target attainment than single-dose depot or intermittent megadose strategies. Intramuscular therapy may remain appropriate in selected patients with malabsorption or persistent non-adherence and should be chosen according to the clinical context rather than regarded as a universally interchangeable alternative to repeated oral dosing.

#### Daily, Weekly, and Monthly Oral Schedules

Daily oral cholecalciferol generally allows more flexible dose adjustment and biochemical reassessment than large intermittent or depot regimens. Oral vitamin D undergoes enteric absorption and hepatic 25-hydroxylation without relying on depot release from muscle or adipose tissue. Comparative pharmacokinetic studies have reported a stronger initial response with oral than intramuscular administration and a higher proportion of patients reaching target concentrations during the loading phase. Clinical support for daily dosing is based primarily on guideline preference, pharmacokinetic considerations, and subgroup signals rather than definitive superiority across all clinical outcomes [48,77,78].

The longer circulating half-life of 25(OH)D means that regular weekly or monthly administration can also maintain vitamin D status when the cumulative dose is equivalent. Randomised comparisons support similar biochemical efficacy for 1000 IU/day, 7000 IU/week, and 30,000 IU/month, and for 1500 IU/day, 10,500 IU/week, and 45,000 IU every 28 days [81,82]. Weekly or monthly schedules may therefore be useful when they improve adherence, simplify supervised administration, or match available formulations. Daily dosing remains advantageous when fine dose adjustment, rapid cessation, or close biochemical titration is required.

These schedules should be described as daily-equivalent replacement rather than as bolus therapy. A monthly dose approximating 30,000 IU corresponds to about 1000 IU/day, whereas 60,000 IU/month corresponds to about 2000 IU/day mathematically but may produce larger short-term peaks and requires caution in frail or fall-prone older adults [83]. Very large monthly, quarterly, or annual doses should not be assumed to reproduce the biological or clinical effects of regular daily, weekly, or modest monthly replacement [5,77,84].

### 10.4. Monitoring, Maintenance, and Inadequate Response

Because serum 25(OH)D changes over several weeks, biochemical reassessment is most informative after an appropriate interval when repeat testing is clinically indicated. Repeat 25(OH)D measurement is not a universal requirement after supplementation; it is most useful when treatment was initiated for documented deficiency, when adherence or absorption is uncertain, when higher-dose therapy is used, or when the result will change ongoing management. Serum calcium and renal function are particularly relevant in patients at risk of disordered mineral metabolism [5,71,73,74,75].

If 25(OH)D fails to rise as expected, clinicians should first evaluate adherence, product potency, formulation, interacting medications, body size, and malabsorption rather than automatically escalating to very large doses. Maintenance requirements should be individualised according to the underlying indication and current condition-specific guidance; older fixed-dose multipliers for obesity or malabsorption should not be applied as universal contemporary rules [77,79,80]. The resulting risk-stratified considerations are summarized in Table 4.

## 11. Public-Health Implications and Policy

The Korean experience illustrates how environmental, behavioural, dietary, and demographic pressures can converge to sustain low population 25(OH)D concentrations. KNHANES data indicate that serum 25(OH)D concentrations declined across the population between 2008 and 2014, against a backdrop of limited national food fortification, low dietary vitamin D intake, incomplete food-composition data for vitamin D, urbanisation, photoprotective behaviour, and population ageing [2,3].

Several public-health considerations emerge from this evidence and should be interpreted as risk-stratified measures that complement, rather than contradict, the 2024 Endocrine Society guideline:

Culturally-adapted food fortification. Population-level fortification may be considered where dietary vitamin D intake is persistently low, but its adoption requires assessment of population coverage, baseline intake, effectiveness, safety, feasibility, cost-effectiveness, and the existing national regulatory context. In Korea, where fortification is less extensive than in some North American settings, these issues remain a policy and research consideration rather than a prescriptive recommendation [3].

**Clinically indicated assessment in high-risk individuals.** Routine population screening is not supported. Measurement of serum 25(OH)D may nevertheless be appropriate when the result is expected to influence management, including suspected osteomalacia, malabsorption, severe dietary restriction, unexplained proximal muscle weakness, selected metabolic bone disorders, or osteoporosis care requiring confirmation of vitamin D adequacy [5].

When deficiency is confirmed, public-health identification should connect to a defined clinical pathway that includes an appropriate oral repletion schedule, maintenance dosing, and selective reassessment rather than ending with diagnosis alone.

**Ongoing evaluation of dietary reference frameworks.** Dietary reference frameworks should continue to be evaluated against contemporary data on dietary intake, fortification coverage, population ageing, obesity, and constrained UVB exposure. However, any revision should be based on population-level benefit–risk assessment rather than on advocacy for higher serum targets.

Environmental and educational measures. Public-health education should distinguish ordinary incidental outdoor activity from intentional ultraviolet exposure. Intentional UV exposure should not be promoted as a primary treatment for vitamin D deficiency because skin-cancer and photodamage risks must remain central to counselling. Air-quality improvement has broad health benefits and may indirectly influence outdoor activity and ground-level UVB availability, but it should not be framed primarily as a vitamin D intervention [15,19].

## 12. Limitations and Research Priorities

This review has several limitations. First, although a focused literature search and explicit evidence-prioritisation approach were used, the narrative design does not guarantee exhaustive study identification and may be affected by source-selection bias. A prospective deduplicated screening log was not maintained, duplicate screening was not performed for every search output, and no formal risk-of-bias tool or certainty-grading framework was applied. Second, the review relies partly on secondary literature and selectively emphasises Korean and East-Asian evidence for regional context, which may limit global generalisability. Third, the evidence spans clinical domains with markedly different levels of causal support, and the interpretive framework necessarily reflects author synthesis. Fourth, substantial heterogeneity exists in baseline vitamin D status, assay methods, dosing regimens, achieved 25(OH)D concentrations, adiposity, adherence, calcium intake, coexisting disease, and clinical endpoints. Finally, evidence that daily, weekly, and monthly schedules produce similar 25(OH)D responses is principally biochemical and short term, and direct randomised evidence comparing alternative serum targets in patients with documented deficiency remains limited. Future trials should enrol clearly characterised deficient populations, report achieved 25(OH)D concentrations and safety outcomes, and evaluate clinically meaningful endpoints.

## 13. Conclusions

Vitamin D deficiency remains a clinically relevant nutritional condition, with its strongest established importance in skeletal mineralisation, osteomalacia, muscle function, and the supportive management of patients with osteoporosis or other clear deficiency-related indications. In contrast, routine vitamin D supplementation of broadly unselected, generally healthy adults has not consistently prevented fractures, falls, cardiovascular events, cancer incidence, or other major extra-skeletal outcomes. Extra-skeletal associations should therefore be interpreted according to the strength and design of the supporting evidence rather than used as stand-alone treatment indications.

The central clinical distinction is between population-wide supplementation for disease prevention and clinically indicated management of established deficiency. The latter should follow current condition-specific or regional guidance, with historical regimens clearly identified as historical, selective biochemical reassessment when it will influence management, and avoidance of very large intermittent boluses in vulnerable populations. Current evidence does not support universal serum 25(OH)D targets above conventional skeletal adequacy thresholds or vitamin D as a primary treatment for cardiovascular, oncological, psychiatric, sleep, or nonspecific pain outcomes.

## Figures and Tables

**Table 1:** Contrasting current, historical, and expert positions on serum 25(OH)D interpretation and testing. The 2011 Endocrine Society document is shown for historical context and is explicitly identified as having been replaced by the 2024 guideline; current prevention guidance and contemporary consensus are distinguished from historical thresholds [5,68,69,70,71].

Source and Year	Position on Target and Testing
Institute of Medicine (2011)	20 ng/mL adequate for skeletal health; basis for most national dietary reference intakes.
Endocrine Society (2011; historical, replaced)	Historically defined deficiency <20 ng/mL (50 nmol/L) and sufficiency ≥30 ng/mL (75 nmol/L), with explicit treatment regimens. The 2024 guideline formally replaces this document.
Endocrine Society (2024)	Current prevention-focused guideline. Advises against routine 25(OH)D screening in generally healthy populations without an established indication and does not endorse a universal 30 ng/mL (75 nmol/L) target.
International consensus statement (2024)	Serum 25(OH)D is the accepted biomarker of vitamin D status, but the interpretation of an optimal concentration depends on the clinical question, population risk, assay context, and intended outcome.

**Table 2:** Contextual interpretation of serum 25(OH)D. Concentrations above conventional skeletal adequacy thresholds are presented descriptively rather than as validated incremental therapeutic categories. Current guidance does not endorse a universal 30 ng/mL (75 nmol/L) target for generally healthy adults, and additional benefit at higher concentrations has not been consistently demonstrated.

25(OH)D Context	Evidence-Based Interpretation
<10 ng/mL (<25 nmol/L)	Markedly low concentration; compatible with severe deficiency and increased risk of rickets/osteomalacia when supported by clinical and biochemical findings.
10–20 ng/mL (25–50 nmol/L)	Low concentration associated with greater skeletal vulnerability; interpretation should consider symptoms, calcium–phosphate metabolism, and the clinical indication for testing.
≥20 ng/mL (≥50 nmol/L)	Compatible with skeletal adequacy for most individuals under the IOM/NASEM framework. Current Endocrine Society guidance does not define a universal higher target for disease prevention.
Above conventional skeletal adequacy thresholds	Higher concentrations, including the historical ≥30 ng/mL (≥75 nmol/L) threshold and ranges proposed in selected higher-target literature, should not be treated as validated incremental therapeutic goals; additional clinical benefit remains unproven.
Markedly elevated concentration, particularly >150 ng/mL (>375 nmol/L) with hypercalcaemia	Interpret with calcium, renal function, symptoms, dose exposure, and potential secondary causes; evaluate for vitamin D toxicity when the biochemical and clinical context is compatible.

**Table 3:** Comparison of adult vitamin D dosing frameworks by source status and clinical purpose. Dietary reference intake, osteoporosis maintenance, regional expert consensus, historical guidance, and current prevention guidance are intentionally distinguished rather than combined as equivalent treatment recommendations [5,16,68,69,79].

Source or Clinical Context	Population and Intent	Adult Oral Dose	Interpretive Point
IOM/NASEM dietary reference framework	Generally healthy adults; population adequacy	600 IU/day at 19–70 years; 800 IU/day above 70 years	Dietary reference intake, not a treatment regimen for documented deficiency.
Korean osteoporosis position statement	Adults requiring skeletal-health support	800–1000 IU/day	Maintenance-oriented recommendation in osteoporosis and fracture-risk care.
Osteoporosis-oriented international guidance	Older adults or patients with osteoporosis	Usually 800–1000 IU/day	Often combined with adequate calcium intake and osteoporosis-specific therapy.
Central and Eastern European expert consensus	Adults seeking sufficient status; selected high-risk groups	800–2000 IU/day; when rapid correction is clinically indicated, 6000 IU/day for 4–12 weeks, followed by 800–2000 IU/day maintenance	Allows a time-limited higher initial dose when rapid correction is clinically indicated, followed by maintenance dosing individualised according to risk.
Endocrine Society guideline (2011; historical, formally replaced)	Adults with documented deficiency	Historically: 6000 IU/day or 50,000 IU/week of vitamin D2 or D3 for 8 weeks; then maintenance 1500–2000 IU/day	Historical therapeutic framework retained for context; it should not be presented as the current Endocrine Society treatment standard.
Endocrine Society guideline (2024; current)	Generally healthy populations and selected empiric-supplementation groups	Use age-specific dietary reference intakes in adults below 75 without another indication; daily lower-dose preferred when treatment is indicated at age ≥ 50 years	Replaces the 2011 guideline; prevention-focused and does not establish a universal serum treatment target for established deficiency.

**Table 4:** Risk-stratified clinical considerations for vitamin D assessment and management in adults. This table is a narrative synthesis, not a prescriptive algorithm. Historical guidance is identified as such, current formal guidance is distinguished from expert consensus, and reassessment is selective rather than universally mandated [5,16,68,69,71,72,73,74,75,77,79].

Clinical Scenario	Assessment	Common Oral Approach	Reassessment and Cautions
Generally healthy adult without a treatment indication	No routine 25(OH)D testing; assess dietary intake and major clinical risk factors	Meet age-specific dietary reference intake, usually 600–800 IU/day from food and/or supplements	Avoid empiric high-dose treatment and routine serial testing.
Older, institutionalised, osteoporosis-treated, or persistently UVB-constrained adult without confirmed severe deficiency	Test when the result will change management; review calcium intake, falls, fracture risk, and medications	Usually 800–1000 IU/day; 800–2000 IU/day or equivalent weekly/monthly dosing may be used according to local guidance and risk	Daily dosing is preferred for frail adults when practical; modest weekly/monthly equivalents may improve adherence.
Documented low 25(OH)D with a clinical indication for treatment, or suspected osteomalacia	Check calcium and renal function; consider phosphate, alkaline phosphatase, and parathyroid hormone	Use condition-specific or contemporary regional guidance. The replaced 2011 Endocrine Society guideline historically used 6000 IU/day or 50,000 IU/week of vitamin D2 or D3 for 8 weeks; contemporary expert consensus also describes time-limited higher-dose repletion in selected patients.	Repeat 25(OH)D and calcium selectively when the result will affect ongoing management, commonly after an interval sufficient to assess response; investigate adherence, absorption, or secondary causes if response is inadequate.
Obesity, malabsorption, bariatric surgery, or medications increasing vitamin D catabolism	Confirm indication and consider specialist input; review formulation and absorption	May require an individualised dose or alternative formulation according to the underlying condition and contemporary condition-specific guidance; avoid automatic use of a fixed two- to three-fold multiplier.	Use biochemical monitoring; avoid automatic escalation without confirming adherence and excluding secondary causes.
Hypercalcaemia, granulomatous disease, lymphoma, primary hyperparathyroidism, advanced kidney disease, or recurrent stones	Specialist evaluation; assess calcium-phosphate balance, renal function, parathyroid hormone, and urinary calcium when indicated	Do not use routine empiric high-dose repletion; dose and formulation depend on the underlying disorder	Monitor closely for hypercalcaemia, hypercalciuria, nephrocalcinosis, and renal deterioration.

## Data Availability

No new data were created or analyzed in this study. Data sharing is not applicable to this article.

## Abbreviations

25(OH)D, 25-hydroxyvitamin D; BMI, body mass index; COVID-19, coronavirus disease 2019; IOM, Institute of Medicine; KNHANES, Korea National Health and Nutrition Examination Survey; PM, particulate matter; SPF, sun protection factor; UVB, ultraviolet-B.

## References

[1] Holick M.F. (2007). Vitamin D deficiency. N. Engl. J. Med..

[2] Park J.H., Hong I.Y., Chung J.W., Choi H.S. (2018). Vitamin D status in South Korean population: Seven-year trend from the KNHANES. Medicine.

[3] Kim K.N., Lee J.S., Shim J.S., Yoon M.O., Lee H.S. (2023). Estimated dietary vitamin D intake and major vitamin D food sources of Koreans: Based on the Korea National Health and Nutrition Examination Survey 2016–2019. Nutr. Res. Pract..

[4] Manson J.E., Cook N.R., Lee I.M., Christen W., Bassuk S.S., Mora S., Gibson H., Gordon D., Copeland T., D’Agostino D. (2019). Vitamin D Supplements and Prevention of Cancer and Cardiovascular Disease. N. Engl. J. Med..

[5] Demay M.B., Pittas A.G., Bikle D.D., Diab D.L., Kiely M.E., Lazaretti-Castro M., Lips P., Mitchell D.M., Murad M.H., Powers S. (2024). Vitamin D for the Prevention of Disease: An Endocrine Society Clinical Practice Guideline. J. Clin. Endocrinol. Metab..

[6] Massé O., Mercurio C.M., Dupuis S., Al Sahwi M., Arruda A., Dallaire G., Desforges K., Dugré N., Williamson D. (2026). Calcium, vitamin D, or combined supplementation to prevent fractures and falls: Systematic review and meta-analysis. BMJ.

[7] Joh H.K., Lim C.S., Cho B. (2015). Lifestyle and Dietary Factors Associated with Serum 25-Hydroxyvitamin D Levels in Korean Young Adults. J. Korean Med. Sci..

[8] Yeum K.J., Song B.C., Joo N.S. (2016). Impact of Geographic Location on Vitamin D Status and Bone Mineral Density. Int. J. Environ. Res. Public Health.

[9] Cashman K.D., Dowling K.G., Škrabáková Z., Gonzalez-Gross M., Valtueña J., De Henauw S., Moreno L., Damsgaard C.T., Michaelsen K.F., Mølgaard C. (2016). Vitamin D deficiency in Europe: Pandemic?. Am. J. Clin. Nutr..

[10] Weiler H.A., Sarafin K., Martineau C., Daoust J.L., Esslinger K., Greene-Finestone L.S., Loukine L., Dorais V. (2023). Vitamin D Status of People 3 to 79 Years of Age from the Canadian Health Measures Survey 2012–2019. J. Nutr..

[11] Alkhaldy A.A., Aljaadi A.M., Jalil A.M.M., Alyoubi D.A., Saleemani H.H., Eid R.H., Almohmadi N.H., Al-Otaibi H.H., Ajabnoor S.M. (2024). Cross-continental national nutrition surveys: A narrative review. BMC Nutr..

[12] Paul S., Kaushik R., Chawla P., Upadhyay S., Rawat D., Akhtar A. (2024). Vitamin-D as a multifunctional molecule for overall well-being: An integrative review. Clin. Nutr. ESPEN.

[13] Baethge C., Goldbeck-Wood S., Mertens S. (2019). SANRA-a scale for the quality assessment of narrative review articles. Res. Integr. Peer Rev..

[14] Ferrari R. (2015). Writing narrative style literature reviews. Med. Writ..

[15] Mousavi S.E., Amini H., Heydarpour P., Amini Chermahini F., Godderis L. (2019). Air pollution, environmental chemicals, and smoking may trigger vitamin D deficiency: Evidence and potential mechanisms. Environ. Int..

[16] Han A., Park Y., Lee Y.K., Park S.Y., Park C.Y. (2022). Position Statement: Vitamin D Intake to Prevent Osteoporosis and Fracture in Adults. J. Bone Metab..

[17] Park H.Y., Lim Y.H., Park J.B., Rhie J., Lee S.J. (2020). Environmental and Occupation Factors Associated with Vitamin D Deficiency in Korean Adults: The Korea National Health and Nutrition Examination Survey (KNHANES) 2010–2014. Int. J. Environ. Res. Public Health.

[18] Breakell T., Kowalski I., Foerster Y., Kramer R., Erdmann M., Berking C., Heppt M.V. (2024). Ultraviolet Filters: Dissecting Current Facts and Myths. J. Clin. Med..

[19] Passeron T., Bouillon R., Callender V., Cestari T., Diepgen T., Green A., van der Pols J., Bernard B., Ly F., Bernerd F. (2019). Sunscreen photoprotection and vitamin D status. Br. J. Dermatol..

[20] Sowah D., Fan X., Dennett L., Hagtvedt R., Straube S. (2017). Vitamin D levels and deficiency with different occupations: A systematic review. BMC Public Health.

[21] Ceglia L. (2009). Vitamin D and its role in skeletal muscle. Curr. Opin. Clin. Nutr. Metab. Care.

[22] Girgis C.M., Clifton-Bligh R.J., Hamrick M.W., Holick M.F., Gunton J.E. (2013). The roles of vitamin D in skeletal muscle: Form, function, and metabolism. Endocr. Rev..

[23] Bollen S.E., Bass J.J., Fujita S., Wilkinson D., Hewison M., Atherton P.J. (2022). The Vitamin D/Vitamin D receptor (VDR) axis in muscle atrophy and sarcopenia. Cell Signal.

[24] Rosendahl-Riise H., Spielau U., Ranhoff A.H., Gudbrandsen O.A., Dierkes J. (2017). Vitamin D supplementation and its influence on muscle strength and mobility in community-dwelling older persons: A systematic review and meta-analysis. J. Hum. Nutr. Diet..

[25] Jang D.G., Ryu S.Y., Park J., Choi S.W. (2020). Low Muscle Mass Is Associated with Lower 25-Hydroxyvitamin D Level in All Age Groups of South Korean Adults: The 2009-2010 Korea National Health and Nutrition Examination Surveys (KNHANES). J. Nutr. Sci. Vitaminol..

[26] Shipton E.E., Shipton E.A. (2015). Vitamin D Deficiency and Pain: Clinical Evidence of Low Levels of Vitamin D and Supplementation in Chronic Pain States. Pain Ther..

[27] Wu Z., Malihi Z., Stewart A.W., Lawes C.M., Scragg R. (2018). The association between vitamin D concentration and pain: A systematic review and meta-analysis. Public Health Nutr..

[28] Xie Y., Farrell S.F., Armfield N., Sterling M. (2024). Serum Vitamin D and Chronic Musculoskeletal Pain: A Cross-Sectional Study of 349,221 Adults in the UK. J. Pain.

[29] Yoo K.O., Kim M.J., Ly S.Y. (2019). Association between vitamin D intake and bone mineral density in Koreans aged ≥ 50 years: Analysis of the 2009 Korea National Health and Nutrition Examination Survey using a newly established vitamin D database. Nutr. Res. Pract..

[30] You H., Shin H.R., Song S., Ly S.Y. (2022). Vitamin D intake and bone mineral density in Korean adults: Analysis of the 2009-2011 Korea National Health and Nutrition Examination Survey. Nutr. Res. Pract..

[31] Hong K.W., Kim M., Lee I., Choi J.E., Shin H.Y. (2026). Real-World Assessment of Osteoporosis-Related Polymorphisms: Negative Findings for Osteoporosis and an Exploratory Association with Vitamin D. Life.

[32] Cianferotti L. (2022). Osteomalacia Is Not a Single Disease. Int. J. Mol. Sci..

[33] Garip Y., Dedeoglu M., Bodur H. (2014). Osteomalacia mimicking spondyloarthropathy: A case report. Osteoporos. Int..

[34] Hereford T., Kellish A., Samora J.B., Reid Nichols L. (2024). Understanding the importance of peak bone mass. J. Pediatr. Soc. N. Am..

[35] Downey C., Kelly M., Quinlan J.F. (2019). Changing trends in the mortality rate at 1-year post hip fracture—A systematic review. World J. Orthop..

[36] Bernstein J., Lee A., Ahn J. (2024). When does annual geriatric hip fracture mortality revert to baseline?. Front. Surg..

[37] Hiemstra T.F., Lim K., Thadhani R., Manson J.E. (2019). Vitamin D and Atherosclerotic Cardiovascular Disease. J. Clin. Endocrinol. Metab..

[38] Nardin M., Verdoia M., Nardin S., Cao D., Chiarito M., Kedhi E., Galasso G., Condorelli G., De Luca G. (2024). Vitamin D and Cardiovascular Diseases: From Physiology to Pathophysiology and Outcomes. Biomedicines.

[39] Rasouli M.A., Darvishzadehdaledari S., Alizadeh Z., Moradi G., Gholami F., Mahmoudian A. (2023). Vitamin D Supplementation and Cardiovascular Disease Risks in More Than 134000 Individuals in 29 Randomized Clinical Trials and 157000 Individuals in 30 Prospective Cohort Studies: An Updated Systematic Review and Meta-analysis. J. Res. Health Sci..

[40] Manson J.E., Bassuk S.S., Buring J.E., Vital Research Group (2020). Principal results of the VITamin D and OmegA-3 TriaL (VITAL) and updated meta-analyses of relevant vitamin D trials. J. Steroid Biochem. Mol. Biol..

[41] Zhou A., Selvanayagam J.B., Hypponen E. (2022). Non-linear Mendelian randomization analyses support a role for vitamin D deficiency in cardiovascular disease risk. Eur. Heart J..

[42] Zhou A., Selvanayagam J.B., Hyppönen E. (2025). RETRACTED: Non-linear Mendelian randomization analyses support a role for vitamin D deficiency in cardiovascular disease risk. Eur. Heart J..

[43] Davey Smith G. (2024). Non-linear Mendelian randomization publications on vitamin D report spurious findings and require major correction. Eur. Heart J..

[44] Charoenngam N., Holick M.F. (2020). Immunologic Effects of Vitamin D on Human Health and Disease. Nutrients.

[45] Munoz A., Grant W.B. (2022). Vitamin D and Cancer: An Historical Overview of the Epidemiology and Mechanisms. Nutrients.

[46] Arayici M.E., Basbinar Y., Ellidokuz H. (2023). Vitamin D Intake, Serum 25-Hydroxyvitamin-D (25(OH)D) Levels, and Cancer Risk: A Comprehensive Meta-Meta-Analysis Including Meta-Analyses of Randomized Controlled Trials and Observational Epidemiological Studies. Nutrients.

[47] Carlberg C., Munoz A. (2022). An update on vitamin D signaling and cancer. Semin. Cancer Biol..

[48] Kuznia S., Zhu A., Akutsu T., Buring J.E., Camargo C.A., Cook N.R., Chen L.-J., Cheng T.-Y.D., Hantunen S., Lee I.-M. (2023). Efficacy of vitamin D(3) supplementation on cancer mortality: Systematic review and individual patient data meta-analysis of randomised controlled trials. Ageing Res. Rev..

[49] Jolliffe D.A., Camargo C.A., Sluyter J.D., Aglipay M., Aloia J.F., Bergman P., Bischoff-Ferrari H.A., Borzutzky A., Bubes V.Y., Damsgaard C.T. (2025). Vitamin D supplementation to prevent acute respiratory infections: Systematic review and meta-analysis of stratified aggregate data. Lancet Diabetes Endocrinol..

[50] Jolliffe D.A., Camargo C.A., Sluyter J.D., Aglipay M., Aloia J.F., Ganmaa D., Bergman P., Bischoff-Ferrari H.A., Borzutzky A., Damsgaard C.T. (2021). Vitamin D supplementation to prevent acute respiratory infections: A systematic review and meta-analysis of aggregate data from randomised controlled trials. Lancet Diabetes Endocrinol..

[51] Entrenas Castillo M.E., Entrenas Costa L.M.E., Vaquero Barrios J.M.V., Alcalá Díaz J.F.A., López Miranda J.L., Bouillon R., Quesada Gomez J.M.Q. (2020). Effect of calcifediol treatment and best available therapy versus best available therapy on intensive care unit admission and mortality among patients hospitalized for COVID-19: A pilot randomized clinical study. J. Steroid Biochem. Mol. Biol..

[52] Zhu L., Zhang Y., Li X., Zou X., Bing P., Qi M., He B. (2025). Vitamin D supplementation for managing COVID-19 in patients with vitamin D deficiency: A systematic review and meta-analysis of randomised controlled trials. BMJ Open.

[53] MMurai I.H., Fernandes A.L., Sales L.P., Pinto A.J., Goessler K.F., Duran C.S.C., Silva C.B.R., Franco A.S., Macedo M.B., Dalmolin H.H.H. (2021). Effect of a Single High Dose of Vitamin D3 on Hospital Length of Stay in Patients With Moderate to Severe COVID-19: A Randomized Clinical Trial. JAMA.

[54] Jolliffe D.A., Holt H., Greenig M., Talaei M., Perdek N., Pfeffer P., Vivaldi G., Maltby S., Symons J., Barlow N.L. (2022). Effect of a test-and-treat approach to vitamin D supplementation on risk of all cause acute respiratory tract infection and COVID-19: Phase 3 randomised controlled trial (CORONAVIT). BMJ.

[55] Menon V., Kar S.K., Suthar N., Nebhinani N. (2020). Vitamin D and Depression: A Critical Appraisal of the Evidence and Future Directions. Indian J. Psychol. Med..

[56] Yao L., Chen M., Zhang N., Ma S., Xie X., Xu S., Nie Z., Wang W., Zhou E., Xu S. (2023). The Mediation Role of Sleep Disturbances between Vitamin D and Depressive Symptoms: A Cross-Sectional Study. Brain Sci..

[57] Xie F., Huang T., Lou D., Fu R., Ni C., Hong J., Ruan L. (2022). Effect of vitamin D supplementation on the incidence and prognosis of depression: An updated meta-analysis based on randomized controlled trials. Front. Public Health.

[58] Cheng Y.C., Huang Y.C., Huang W.L. (2020). The effect of vitamin D supplement on negative emotions: A systematic review and meta-analysis. Depress. Anxiety.

[59] Arabshahi V., Khoddami M., Milajerdi M., Moabedi M., Milajerdi A. (2024). Association between dietary intake of vitamin D and risk of depression, anxiety, and sleep disorders among physically active adults: A cross-sectional study. Front. Nutr..

[60] Gao Q., Kou T., Zhuang B., Ren Y., Dong X., Wang Q. (2018). The Association between Vitamin D Deficiency and Sleep Disorders: A Systematic Review and Meta-Analysis. Nutrients.

[61] Abboud M. (2022). Vitamin D Supplementation and Sleep: A Systematic Review and Meta-Analysis of Intervention Studies. Nutrients.

[62] Finan P.H., Goodin B.R., Smith M.T. (2013). The association of sleep and pain: An update and a path forward. J. Pain.

[63] Neale R.E., Baxter C., Romero B.D., McLeod D.S.A., English D.R., Armstrong B.K., Ebeling P.R., Hartel G., Kimlin M.G., O’Connell R. (2022). The D-Health Trial: A randomised controlled trial of the effect of vitamin D on mortality. Lancet Diabetes Endocrinol..

[64] Scragg R., Stewart A.W., Waayer D., Lawes C.M.M., Toop L., Sluyter J., Murphy J., Khaw K.-T., Camargo C.A. (2017). Effect of Monthly High-Dose Vitamin D Supplementation on Cardiovascular Disease in the Vitamin D Assessment Study: A Randomized Clinical Trial. JAMA Cardiol..

[65] Wortsman J., Matsuoka L.Y., Chen T.C., Lu Z., Holick M.F. (2000). Decreased bioavailability of vitamin D in obesity. Am. J. Clin. Nutr..

[66] Drincic A.T., Armas L.A., Van Diest E.E., Heaney R.P. (2012). Volumetric dilution, rather than sequestration best explains the low vitamin D status of obesity. Obesity.

[67] Larysz D., Recław R., Suchanecka A., Dziurawiec W., Tkacz R., Strońska-Pluta A., Chmielowiec K., Grzywacz A., Chmielowiec J. (2025). Vitamin D Genetics Beyond Serum 25(OH)D: VDR rs2228570 (FokI) Polymorphism, Inflammation, and Quality of Life in Orthopedic Patients. Nutrients.

[68] Ross A.C., Manson J.E., Abrams S.A., Aloia J.F., Brannon P.M., Clinton S.K., Durazo-Arvizu R.A., Gallagher J.C., Gallo R.L., Jones G. (2011). The 2011 report on dietary reference intakes for calcium and vitamin D from the Institute of Medicine: What clinicians need to know. J. Clin. Endocrinol. Metab..

[69] Holick M.F., Binkley N.C., Bischoff-Ferrari H.A., Gordon C.M., Hanley D.A., Heaney R.P., Murad M.H., Weaver C.M. (2011). Evaluation, treatment, and prevention of vitamin D deficiency: An Endocrine Society clinical practice guideline. J. Clin. Endocrinol. Metab..

[70] Demay M.B., Pittas A.G., Bikle D.D., Diab D.L., Kiely M.E., Lazaretti-Castro M., Lips P., Mitchell D.M., Murad M.H., Powers S. (2024). Vitamin D for the Prevention of Disease: An Endocrine Society Clinical Practice Guideline. J. Clin. Endocrinol. Metab..

[71] Giustina A., Bilezikian J.P., Adler R.A., Banfi G., Bikle D.D., Binkley N.C., Bollerslev J., Bouillon R., Brandi M.L., Casanueva F.F. (2024). Consensus Statement on Vitamin D Status Assessment and Supplementation: Whys, Whens, and Hows. Endocr. Rev..

[72] Wimalawansa S.J. (2025). Vitamin D’s Impact on Cancer Incidence and Mortality: A Systematic Review. Nutrients.

[73] Tebben P.J., Singh R.J., Kumar R. (2016). Vitamin D-Mediated Hypercalcemia: Mechanisms, Diagnosis, and Treatment. Endocr. Rev..

[74] Galior K., Grebe S., Singh R. (2018). Development of Vitamin D Toxicity from Overcorrection of Vitamin D Deficiency: A Review of Case Reports. Nutrients.

[75] Berger M.M., Shenkin A., Dizdar O.S., Amrein K., Augsburger M., Biesalski H.-K., Bischoff S.C., Casaer M.P., Gundogan K., Lepp H.-L. (2024). ESPEN practical short micronutrient guideline. Clin. Nutr..

[76] Li X., Liu Y., Chen X., Reichetzeder C., Elitok S., Krämer B.K., Hocher B. (2024). Target Values for 25-Hydroxy and 1,25-Dihydroxy Vitamin D Based on Their Associations with Inflammation and Calcium-Phosphate Metabolism. Nutrients.

[77] Bilezikian J.P., Formenti A.M., Adler R.A., Binkley N., Bouillon R., Lazaretti-Castro M., Marcocci C., Napoli N., Rizzoli R., Giustina A. (2021). Vitamin D: Dosing, levels, form, and route of administration: Does one approach fit all?. Rev. Endocr. Metab. Disord..

[78] Wylon K., Drozdenko G., Krannich A., Heine G., Dolle S., Worm M. (2017). Pharmacokinetic Evaluation of a Single Intramuscular High Dose versus an Oral Long-Term Supplementation of Cholecalciferol. PLoS ONE.

[79] Pludowski P., Takacs I., Boyanov M., Belaya Z., Diaconu C.C., Mokhort T., Zherdova N., Rasa I., Payer J., Pilz S. (2022). Clinical Practice in the Prevention, Diagnosis and Treatment of Vitamin D Deficiency: A Central and Eastern European Expert Consensus Statement. Nutrients.

[80] Giustina A., di Filippo L., Facciorusso A., Adler R.A., Binkley N., Bollerslev J., Bouillon R., Casanueva F.F., Cavestro G.M., Chakhtoura M. (2023). Vitamin D status and supplementation before and after Bariatric Surgery: Recommendations based on a systematic review and meta-analysis. Rev. Endocr. Metab. Disord..

[81] Takács I., Tóth B.E., Szekeres L., Szabó B., Bakos B., Lakatos P. (2017). Randomized clinical trial to comparing efficacy of daily, weekly and monthly administration of vitamin D(3). Endocrine.

[82] Ish-Shalom S., Segal E., Salganik T., Raz B., Bromberg I.L., Vieth R. (2008). Comparison of daily, weekly, and monthly vitamin D3 in ethanol dosing protocols for two months in elderly hip fracture patients. J. Clin. Endocrinol. Metab..

[83] Bischoff-Ferrari H.A., Dawson-Hughes B., Orav E.J., Staehelin H.B., Meyer O.W., Theiler R., Dick W., Willett W.C., Egli A. (2016). Monthly High-Dose Vitamin D Treatment for the Prevention of Functional Decline: A Randomized Clinical Trial. JAMA Intern. Med..

[84] Sanders K.M., Stuart A.L., Williamson E.J., Simpson J.A., Kotowicz M.A., Young D., Nicholson G.C. (2010). Annual high-dose oral vitamin D and falls and fractures in older women: A randomized controlled trial. JAMA.

